# Bioaccessibility of Macrominerals and Trace Elements from Tomato (*Solanum lycopersicum* L.) Farmers’ Varieties

**DOI:** 10.3390/foods11131968

**Published:** 2022-07-02

**Authors:** María Ciudad-Mulero, José Pinela, Ana Maria Carvalho, Lillian Barros, Virginia Fernández-Ruiz, Isabel C. F. R. Ferreira, María de Cortes Sánchez-Mata, Patricia Morales

**Affiliations:** 1 Dpto. Nutrición y Ciencia de los Alimentos, Facultad de Farmacia, Universidad Complutense de Madrid (UCM), Pza. Ramón y Cajal, s/n, E-28040 Madrid, Spain; mariaciudad@ucm.es (M.C.-M.); vfernand@farm.ucm.es (V.F.-R.); cortesm@ucm.es (M.d.C.S.-M.); 2 Centro de Investigação de Montanha (CIMO), Instituto Politécnico de Bragança, Campus de Santa Apolónia, 5300-253 Bragança, Portugal; anacarv@ipb.pt (A.M.C.); lillian@ipb.pt (L.B.); iferreira@ipb.pt (I.C.F.R.F.)

**Keywords:** *Solanum lycopersicum* L., local varieties, minerals, micronutrients, in vitro gastrointestinal digestion, bioaccessibility, dietary reference intakes

## Abstract

Traditional farmers’ varieties of tomato grown under extensive farming techniques are considered delicious and healthy foods and are preferred by local consumers. Tomatoes are an important component of a healthy diet, as they provide essential micronutrients, including minerals, which are vital to healthy development, disease prevention, and wellbeing. Given the considerable dietary intake of tomatoes and the scarcity of information about the bioaccessibility of inorganic constituents in this fruit, this study was carried out to evaluate the content and bioaccessibility of minerals (macro- and microelements) in tomato farmers’ varieties widely cultivated in northeastern Portugal homegardens. Among the macroelements, K stood out as the most abundant mineral in the studied varieties, followed by Mg, Ca, and Na. Regarding the microelements, while the yellow tomato had higher concentrations of Fe and Cu, the round tomato had more Zn and Mn. The in vitro bioaccessibility assessment showed that, among the macroelements, Mg was more bioaccessible than Ca and K when all the tomato varieties were considered together. Among the microelements, Cu seemed to be the most bioaccessible. Although the contribution of a 100 g serving of the studied tomato farmers’ varieties to the dietary reference intakes (DRIs) of minerals is relatively low, this food could contribute to reaching these mineral requirements, as it is included in the diet of most of the population, especially in Mediterranean regions.

## 1. Introduction

Tomato (*Solanum lycopersicum* L.; Syn: *Lycopersicon esculentum* Mill.) has been cultivated for over 400 years and, to date, thousands of cultivars have been developed [1]. Through intensive breeding and natural selection activities, scientists, breeders, and farmers from all over the world have created a wide range of cultivars and varieties from the single species *Solanum lycopersicum*. These varieties display different morphological and agronomic characteristics, along with organoleptic or sensory properties that determine their use. In northeastern Portugal, local populations still prefer to consume traditional farmers’ varieties of tomato, which are grown under particular extensive farming techniques and considered delicious and healthy foods [2]. These local varieties resulted from the continuous improvement and selection of desirable features over time by local farmers through a sustainable seed system.

Today, the annual production and consumption of fresh and processed tomatoes varies widely across countries and regions worldwide [3]. Among the European Union (EU) 27, Portugal ranked ninth in tomato production for fresh consumption in 2020 [4]. Despite this, fresh tomato was the horticultural crop with the highest production (144 thousand tons) in Portugal [5]. However, Portuguese statistical data do not specify if local farmer varieties and landraces of tomato contributed to this number. According to a European Commission report [6], the per capita consumption of fresh tomatoes in the EU is expected to remain stable (15 kg) until 2031, with an increase in the consumption of small tomatoes. Tomato is considered the most valuable fruit crop and an important component of a healthy diet, providing essential nutrients such as vitamins, lycopene, minerals, and polyphenols [7,8,9,10,11,12]. The nutrient density of foods is important for achieving an optimal micronutrient status in the human diet. However, many food systems worldwide do not provide enough micronutrients to ensure adequate intakes, and millions of people experience micronutrient deficiencies [13,14].

Minerals are micronutrients that are vital to healthy human development, disease prevention, and wellbeing. According to EU regulations [15,16,17], food products can be labeled with health claims related to minerals when they qualify as sources of certain minerals (providing at least 15% of the nutrient reference values). Examples of these claims, which can be included in the packaging and/or advertising of foods that meet the specific use conditions, include: Potassium (K) contributes to the normal functioning of the nervous system, muscle function, and the maintenance of blood pressure [18]. Calcium (Ca) contributes to normal blood clotting, energy-yielding metabolism, and neurotransmission. It is involved in cell division and specialization and is needed for healthy bones and teeth [19]. Magnesium (Mg) contributes to reducing tiredness and fatigue, the electrolyte balance, muscle function, and protein synthesis. Iron (Fe) plays a central role in metabolic processes involving oxygen transport and storage, as well as oxidative metabolism and cellular growth [20]. Copper (Cu) contributes to the maintenance of connective tissues, nervous and immune system function, Fe transport, skin and hair pigmentation, and protection from oxidative stress [21,22]. Manganese (Mn) contributes to the formation of connective tissue and the protection of cells from oxidative stress [22]. Zinc (Zn) also protects from oxidative stress and contributes to nutrient metabolism, DNA and protein synthesis, normal cognitive function, and the maintenance of the immune system [22].

Therefore, in order to fulfil their dietary reference intake (DRI) of minerals and other nutrients and achieve healthier diets, consumers look for appetizing foods with an adequate nutritional profile, including tomatoes. In addition, the adherence to a varied and balanced diet, rich in micronutrients and bioactive compounds, must be recommended to enhance human health and improve conditions to counter infectious agents, including COVID-19, which constitutes a major health concern worldwide [23].

The contents of certain elements in foods do not always indicate the real nutritional contribution of a diet, as only a portion of the ingested nutrient is absorbed. Thus, bioaccessibility studies are essential to allow a better assessment of the element levels provided by foods. In these studies, the bioaccessible fraction refers to the amount of the element/compound that is released from the food matrix and is soluble in the gastrointestinal tract, making it available for absorption by the intestinal epithelium. Studies on the bioaccessibility of polyphenols and carotenoids are more common in tomatoes [24,25,26,27,28], while the literature on mineral bioaccessibility is still scarce [29]. Furthermore, the mineral content in tomatoes differs between varieties and cultivation methods [30].

Therefore, given the considerable dietary intake of tomatoes and the lack of information about the bioaccessibility of the inorganic constituents of this fruit, this study was carried out to evaluate the content and bioaccessibility of mineral elements in four tomato farmers’ varieties cultivated in northeastern Portugal homegardens through experiments simulating gastrointestinal digestion.

## 2. Materials and Methods

### 2.1. Plant Material

Four tomato farmers’ varieties (*Solanum lycopersicum* L.; syn: *Lycopersicon esculentum* Mill.) cultivated in rural communities from Miranda do Douro, northeastern Portugal, were chosen according the most appreciated characteristics, for instance, agronomic and morphological features and sensory properties, which determine their use [8]. Such varieties are referred to by their local vernacular names and are used differently. The fruits of the variety “tomate amarelo” (yellow tomato), of an intense yellow color even when ripened, are consumed raw in salads; “tomate redondo” (round tomato) is round-shaped like a potato and eaten raw, stewed with fish and meat, or prepared in sauce; “tomate comprido” (long tomato) is similar to plum tomato and is mainly frozen and stored, to be available for cooking during winter; and “tomate coração de boi” (oxheart tomato) is a large, fleshy, juicy, heart-shaped tomato that is mostly consumed raw in traditional summer salads seasoned with olive all and oregano, used for cooking, or used for preparing a traditional marmalade [31,32].

Tomato fruits at the ripening stage were harvested randomly from the middle of six plants of each of the four varieties in selected homegardens of two villages in the studied area. All tomato plants were grown under the same soil and climatic conditions and similar agricultural practices. The seeds were extracted and kept by local farmers. The ripening stage for all samples was determined by visual methods at the full maturity stage of the fruits, established according to local consumers’ criteria, mostly based on fruit color and texture. The edible portion of six fruits of each variety (corresponding to the pericarps without jointed pedicels) was prepared; lyophilized (FreeZone 4.5 model 7750031, Labconco, Kansas, MO, USA; collector chamber at −50 °C and 0.012 torr) until constant weight; reduced to a fine powder; and then mixed to obtain representative samples. The average weight and moisture content of each tomato farmers’ variety is shown in Table 1.

### 2.2. Standards and Reagents

The macro- (Ca, Mg, Na, and K) and trace (Fe, Cu, Mn, and Zn) element standards (CaCO_3_, Mg band, NaCl, KCl, Fe(NO_3_)_3_, Cu(NO_3_)_2_, Mn(NO_3_)_2_, and Zn(NO_3_)_2_; >99% purity), as well as LaCl_2_ and CsCl (>99% purity) were purchased from Merck (Darmstadt, Germany). Pepsin (from porcine stomach mucosa; 1.470 units/mg prot; P-7000); pancreatin (from porcine pancreas, activity equivalent to 4 x U.S.P. specifications; P-1750); and porcine bile (B-8631) were purchased from Sigma-Aldrich Co. (St. Louis, MO, USA). All other reagents were purchased from Panreac Química S.L.U. (Barcelona, Spain). Water was treated in a Milli-Q water purification system (TGI Pure Water Systems, Indianapolis, IN, USA).

### 2.3. Analysis of Mineral Elements

The powdered samples (~200 mg) were mineralized at 550 °C according to method 930.05 of the AOAC procedures for ash (total mineral content) determination. Subsequently, mineral elements were extracted with an acid mixture (2 mL of 50% HCl + 2 mL 50% HNO_3_) and made up to an appropriate volume with distilled water, at which point Fe, Cu, Mn, and Zn were directly measured. For Ca and Mg determination, a dilution with 1.16% La_2_O_3_/HCl (leading to LaCl_2_) was prepared in order to avoid interferences, and a dilution with 0.2% CsCl was prepared for Na and K analysis. Triplicate mineralization and extractions were carried out on the same material. Mineral concentrations were determined in the acid extracts by atomic absorption spectroscopy (AAS) with air/acetylene flame, using Analyst 200 Perkin Elmer equipment (Perkin Elmer, Waltham, MA, USA), as previously described by Ruiz-Rodríguez et al. [33]. The wavelengths used were as follows: 589.0 nm for Na analysis, 769.9 nm for K, 422.7 nm for Ca, 286.2 nm for Mg, 324.8 nm for Cu, 248.3 nm for Fe, 279.5 nm for Mn, and 213.9 nm for Zn. Limit of detection (LOD), limit of quantification (LOQ), linearity, recovery, repeatability, and reproducibility were accepted as previously assessed [34].

### 2.4. Determination of Minerals’ In Vitro Bioaccessibility

The in vitro gastrointestinal model consisted of an initial simulation phase of intraluminal digestion, followed by intestinal absorption using a dialysis model. The minerals’ bioaccessibility was estimated using 25 mL of aqueous solutions prepared from the powdered samples (20 mg/mL) as previously described by Ramírez-Moreno et al. [35]. Gastric digestion was simulated by adjusting the pH of each sample to 2; adding pepsin solution (40 mg/mL of HCl 0.1 M; 150 μL); and incubating the mixture in a water bath at 37 °C for 2 h with stirring (60 osc/min). The intestinal processes were simulated by adding to the digested product a pancreatin/bile solution (5/25 mg of pancreatin/bile per 1 mL of 0.1 M NaHCO_3_). Then, the mixture was transferred to dialysis membranes (Medicell 7000/2, width 34 mm, 7000 MW cut off) previously boiled in distilled water for 15 min, and the dialysis membranes/mixtures were placed into a flask containing 250 mL of NaHCO_3_ pH 7.5 and incubated in a water bath at room temperature for 3 h with stirring (60 osc/min). After dialysis, the obtained final solution of NaHCO_3_ pH 7.5 was frozen and lyophilized for further assays.

Bioaccessibility was calculated as the percentage of the dialyzed mineral with regard to the total content of each mineral in the digested samples.

### 2.5. Statistical Analysis

Analyses were carried out in triplicate, and each repetition was measured three times. Results (*n* = 9) were expressed as mean ± standard deviation. All statistical tests were performed at a 5% significance level using SPSS Statistics software (IBM SPSS Statistics for Windows, Version 22.0, Armonk, NY, USA: IBM Corp.). Differences among samples were analyzed using one-way analysis of variance (ANOVA). The fulfilment of the ANOVA requirements, specifically the normal distribution of residuals and the homogeneity of variance, was assessed by means of the Shapiro–Wilk and Levene tests, respectively. Tukey’s honestly significant difference (HSD) test was used for multiple comparisons.

## 3. Results and Discussion

### 3.1. Mineral Composition

Mineral elements are naturally occurring inorganic solid substances found in tomatoes and other foods that are essential for a variety of bodily functions. The composition of macroelements (Na, K, Ca, and Mg) and trace elements (Fe, Cu, Mn, and Zn) in the tomato farmers’ varieties is shown in Table 2. As expected, K stood out as the most abundant element in the studied tomato varieties, with contents ranging from 158 to 215 mg/100 g fw, in oxheart tomato and yellow tomato, respectively. Mg ranked second with concentrations between 8.9 mg/100 g fw in oxheart tomato and 10 mg/100 g fw in long tomato. These values did not differ significantly (*p* > 0.05) between varieties. The yellow tomato, which had the highest K content, also stood out for its content of Ca (6.8 mg/100 g dw) and Na (3.0 mg/100 g fw), with the concentration of these macroelements being statistically higher (*p* > 0.05) than those found in the other analyzed varieties.

Researchers have previously confirmed that mineral concentrations in tomato fruits are strongly influenced by the genotype [34,36]. However, regardless of the variety, K is the prevalent mineral found in tomatoes [34,36,37,38,39]. Indeed, among the macroelements studied by Rouphael et al. [40], K was by far the most abundant mineral constituent in two different greenhouse tomato cultivars, followed by Ca, P, Mg, and Na. Similar contents of macroelements were found by Bonemann et al. [29], who reported values of K, Mg, and Ca between 217.3 and 377.9, 8.0 and 10.8, and 4.2 and 18.7 mg/100 g fw, respectively, in different tomato cultivars from Brazil. Erika et al. [36] analyzed the mineral profile of twenty tomato cultivars, including cocktail-type and salad-type tomatoes. They reported mean values for K, Mg, and Ca of 285, 12.3, and 12.3 mg/100 g fw, respectively, and observed that the concentration of all analyzed minerals tended to be higher in the cocktail cultivars than the salad tomatoes. In another study, Rosa-Martínez et al. [39] determined the mineral composition of ten varieties of tomato grown under the same environmental conditions using organic agricultural practices. The authors harvested the fruits at the red stage of maturity and reported values of K (68.36–117.6 mg/100 g) and Mg (4.00–9.55 mg/100 g) lower than those found in the present work. The values of Ca (5.97–11.35 mg/100 g) reported by these authors were higher compared to our results. However, they obtained a similar concentration of Na (1.44–2.67 mg/100 g).

Regarding trace elements, while the yellow tomato had higher concentrations of Fe (0.49 mg/100 g fw) and Cu (0.14 mg/100 g fw), the round tomato had more Zn (0.345 mg/100 g fw) and Mn (0.047 mg/100 g fw) (Table 2). On the other hand, oxheart tomato, the farmers’ variety with the lowest levels of Na and K, also contained the lowest concentration of trace elements. Guil-Guerrero and Rebolloso-Fuentes [37] stated that the content of most microelements in tomatoes varies widely, as the microelement profile is strongly influenced by agronomical practices. The authors reported higher values of Fe, Cu, Mn, and Zn than those found in the present work. Comparing our results for the microelement contents in the analyzed tomato farmers’ varieties with the information published in the scientific literature, Rosa-Martínez et al. [39] reported similar values of Zn (0.08–0.18 mg/100 g) but a lower content of Fe (0.11–0.24 mg/100 g) and Cu (0.02–0.06 mg/100 g), whereas Hernández Suárez et al. [30] found lower concentrations of Fe (0.18–0.22 mg/100 g), Cu (0.024–0.032 mg/100 g), and Zn (0.069–0.086 mg/100 g) but a higher amount of Mn (0.054–0.066 mg/100 g). Moreover, the results for Fe (0.129–0.302 mg/100 g) and Zn (0.162–0.336 mg/100 g) obtained by Bonemann et al. [29] were in accordance with those reported in the present study. According to the data obtained in the present work, as well as the bibliographic information, and considering that tomatoes are a widely consumed vegetable, it is possible to state that tomatoes show a favorable mineral profile.

### 3.2. Bioaccessibility of Mineral Elements

Interest in the bioaccessibility of phytochemicals, including mineral elements, has greatly increased due to the existence of micronutrient deficiencies that are related to health concerns [41]. The intestinal absorption of minerals varies depending on the element: Na can be absorbed by an electrical and chemical concentration gradient; K diffuses through channels and into cells by the Na^+^/K^+^ pump; Mg entry from the intestinal lumen occurs by two mechanisms, a transporter-facilitated process and simple diffusion; Ca is absorbed by active or passive transport, depending on its luminal concentration; non-heme Fe, found in foods of plant origin, is absorbed by facilitated diffusion down a concentration gradient; the absorption of Cu is achieved in the short intestine by facilitated diffusion, and it exits by active transport into the bloodstream, where it is bound to ceruloplasmin; Mn reaches enterocytes by active transport; and Zn is absorbed by active or passive transport, depending on its luminal concentration [42]. Table 2 presents the results of the bioaccessibility of minerals from the tomato farmers’ varieties after simulated gastrointestinal digestion. Among the macrominerals, Mg was more bioaccessible (54%) than Ca (18%) and K (16%) when considering the four tomato varieties together. The varieties presented comparable bioaccessibility values for Mg and K, but the values were quite divergent for Ca, ranging from 5.58% in round tomato to 28.77% in long tomato, in which Mg was also more bioaccessible. Among the microelements, Cu seemed to be the most bioaccessible trace element (about 59% bioaccessibility), especially in long tomato (84.85%). On the other hand, round tomato displayed a low bioaccessibility for this element (38.26%), as well as for Mn (30.60%) and Zn (17.47%). High levels of polyphenols, compounds that may have compromised the bioaccessibility [43], have already been described for this tomato variety [8]. We found that Fe did not become bioaccessible for further absorption, as our results showed that this mineral was not present in the solution obtained after the dialysis process. As observed for Ca and Mg, Zn and Mn were highly bioaccessible from oxheart tomato (70.95% and 82.40%, respectively). In general, the bioaccessibility of the three trace elements (Mn, Cu, and Zn) was quite variable depending on the tomato variety under analysis, mainly Mn and Zn, followed by Cu. Although oxheart tomato displayed the lowest levels of trace elements (and Na), Mn, Zn and Ca were found to be quite bioaccessible from this variety, which is commonly consumed cooked and in traditional marmalade. Furthermore, it should be taken into account that the absorption of Mg, Ca, Mn, and Zn is improved by the active-transport mechanisms that take place in the physiological gut barrier.

The bioaccessibility of different minerals could be influenced by the presence of antinutritional factors, such as oxalates, phytic acid, tannins, or saponins [44]. These undesirable components have been found in some varieties of tomato [37,45] and may exist in different concentrations in the four tomato farmers’ varieties under analysis, affecting the bioaccessibility of minerals to different extents. Variations in the composition and firmness of these tomato varieties [8] can also affect their digestibility and, consequently, the bioaccessibility of certain constituents. In particular, the bioaccessibility of Fe, Zn, and Ca could be reduced by phytic acid, as this compound binds different essential macro- and microelements, decreasing their availability for human nutrition. The bioavailability of Fe is also reduced by tannins, whose concentration in plants varies depending on the species. Tomato fruits contain oxalate [46], which is an antinutritional factor that negatively affects the bioaccessibility of minerals, as it forms insoluble salts with Ca^2+^, Fe^2+^, and Mg^2+^, making these minerals unavailable for human nutrition [45]. Kyomugasho et al. [47] investigated the bioaccessibility of Ca and Fe in tomato purées by simulating in vitro digestion. They observed that the bioaccessibility values of Ca were 30.9% (high-pressure-treated tomatoes) and 55.8% (high-temperature-blanched tomatoes). In the case of Fe, the authors obtained bioaccessibility values of 27.9% and 58.3% when tomatoes were treated under high pressure and blanched at a high temperature, respectively. The bioaccessibility percentages found in the cited work were higher than those found in the present study. These differences could be due to the application of high pressure and a high temperature, reducing the antinutritional factors that decrease mineral bioaccessibility [48]. In another study, the dialyzability of Fe and Zn in tomatoes and tomato products produced by conventional or organic farming was measured, and the researchers obtained higher values for Fe (35.06–50.90%) and similar values for Zn (40.70–60.06%) [49] compared with the results obtained in the present work. In addition, the study carried out by Bonemann et al. [29] provided information about the bioaccessible fraction of different minerals in five different tomato cultivars. The values reported by the authors ranged between 36 and 98%, 57 and 96%, 28 and 66%, and 14 and 56 % for Fe, Cu, Mn, and Zn, respectively. These results, with the exception of the Fe bioaccessible fraction, are in accordance with those shown in Table 2.

### 3.3. Contribution to Mineral Requirements

Tomatoes are an important element of the Mediterranean diet and the second most important vegetable crop worldwide, representing a significant part of the human diet [41]. As shown in Table 3, the contribution of a 100 g serving of the studied tomato farmers’ varieties to the dietary reference intakes (DRIs) of minerals (according to European Regulation (EU) No 1169/2011) [16] is relatively low. Cu and K were the elements with higher contents, contributing approximately 12% and 9% of the DRIs of 1 and 2000 mg/day, respectively. The tomato varieties contributed 2.5% of the DRI of Mg (375 mg/day) and less than 2.4% of the DRIs for the following elements, in decreasing order: Fe > Zn > Mn > Ca. The yellow tomato variety contributed the most to the DRIs of K (10.76%), Ca (0.85%), Fe (3.47%), and Cu (13.53%). The largest contributions of Mn (2.36%) and Zn (3.45%) came from round tomato (Table 3).

Although the obtained values did not reach the minimum levels established by the current regulations [15,16,17], meaning that it is not possible advertise nutritional or health claims, tomatoes are a healthy food that contribute to fulfilling mineral intake requirements, as they are included in the diet of most of the population, especially in Mediterranean regions.

According to the World Health Organization (WHO) [51], high Na consumption (>2 g/day, equivalent to 5 g salt/day) and insufficient K intake (less than 3.5 g/day) contribute to high blood pressure and increase the risk of heart disease and stroke. In this sense, the analyzed tomato farmers’ varieties showed a favorable Na/K ratio. As shown Table 3, the yellow tomato variety contributed the most to the recommended Na intake, though it was still far from the stipulated limit.

It is important to note that the contribution of each element to the DRIs discussed above was calculated from the total mineral contents in Table 2, as the current regulations define DRIs and nutrition and health claims based on the total nutrient content of foods. However, if the bioaccessibility of nutrients was considered, the contribution would be lower. As shown in Table 3, the contributions of Cu and K decreased from 12% and 9% to 7% and 1.4%, respectively. For the other elements, the contribution was below 1.5%. Thus, if the bioaccessible fraction is considered, the tomato varieties offer the same overall contribution of K and Mg, given the low bioaccessibility of K (Table 2).

Since the four tomato varieties were of different sizes and therefore different weights (Table 1), their contribution to the DRIs of minerals was also calculated based on their weight (Table 4). Thus, given the higher average weight of oxheart tomato (465 mg), each individual fruit of this variety offers a greater contribution to the DRIs of minerals, followed by the yellow variety.

Although the micronutrient density is important for achieving an optimal nutritional status and fighting malnutrition, it is important to note that the high consumption of tomatoes worldwide makes them an exceptional contributor to DRIs. Thus, even if the mineral contents in the studied tomato farmers’ varieties were not high, their consumption is important for providing minerals and other micronutrients to the local populations who have grown and consumed them for generations. In addition, consumer tendencies show that they prefer local foods, which they believe are healthier and have better organoleptic properties. Some consumers also consider food safety and environmental awareness to be the most important factors in the decision to consume local products generated under extensive farming systems. For this reason, tomato varieties with favorable nutritional profiles, such as those analyzed in the present study, are particularly important.

## 4. Conclusions

Tomatoes are considered the most valuable fruit crop worldwide and are an important component of a healthy diet. They provide essential nutrients, including minerals, which are micronutrients vital to healthy development, disease prevention, and wellbeing. The results obtained in this study showed that four tomato farmers’ varieties widely cultivated in northeastern Portugal homegardens have an interesting mineral profile. Among the macroelements, K stood out as the most abundant mineral in the studied tomato varieties, followed by Mg, Ca, and Na. Regarding the microelements, while the yellow tomato had higher concentrations of Fe and Cu, the round tomato had more Zn and Mn. The results of the in vitro bioaccessibility tests showed that, among the macroelements, Mg was more bioaccessible than Ca and K when considering the all tomato varieties together. Among the microelements, Cu seemed to be the most bioaccessible. Although the contribution of a 100 g serving of the studied tomato farmers’ varieties to the DRIs of minerals was relatively low, this food contributes substantially to fulfilling mineral intake requirements, as it is included in the diet of most of the population, especially in Mediterranean regions. 

The results of this work may have practical applications in the development of tomato-based food formulations with improved mineral bioaccessibility. In the future, it is important to analyze the compounds that affect the bioaccessibility of the studied mineral elements. It would also be interesting to compare the bioaccessibility results of this study with in vivo bioavailability data and to evaluate the molecular mechanisms involved in the bioavailability and bioactivity of minerals upon tomato ingestion. 

## Figures and Tables

**Table 1:** Moisture content and average weight of the tomato farmers’ varieties.

Tomato Farmers’ Variety	Similar Commercial Type	Average Weight (g)	Water Content (%)
Yellow tomato	Yellow tomato	190	90.6
Round tomato	Round standard tomato	116	92.2
Long tomato	Plum tomato	132	93.7
Oxheart tomato	Beefsteak tomato	465	92.8

**Table 2:** Total and in vitro bioaccessible macrominerals and trace elements of four tomato farmers’ varieties.

Macromineral (mg/100 g fw)	Na		K			Ca			Mg		
	Total	Bioaccessible	Total	Bioaccessible	B%	Total	Bioaccessible	B%	Total	Bioaccessible	B%
Yellow tomato	3.0 ± 0.2 ^a^	nd	215 ± 11 ^a^	38 ± 2	17.55	6.8 ± 0.2 ^a^	0.7 ± 0.1	10.25	9.3 ± 0.8	5.2 ± 0.3	56.01
Round tomato	1.32 ± 0.07 ^b^	nd	174 ± 8 ^b^	30 ± 3	17.24	4.4 ± 0.2 ^b^	0.25 ± 0.04	5.58	9.3 ± 0.3	4.5 ± 0.6	48.30
Long tomato	1.14 ± 0.07 ^b^	nd	170 ± 10 ^b^	21 ± 2	12.33	4.5 ± 0.4 ^b^	1.28 ± 0.02	28.77	10 ± 1	5.2 ± 0.2	49.89
Oxheart tomato	0.58 ± 0.07 ^c^	nd	158 ± 5 ^b^	24 ± 2	15.12	4.8 ± 0.2 ^b^	1.30 ± 0.02	27.18	8.9 ± 0.2	5.6 ± 0.2	62.64
One-way ANOVA ^#^	<0.001		<0.001		16 ± 2 *	<0.001		18 ± 10 *	0.337		54 ± 6 *
**Trace element** **(mg/100 g fw)**	**Fe**		**Cu**			**Mn**			**Zn**		
	**Total**	**Bioaccessible**	**Total**	**Bioaccessible**	**B%**	**Total**	**Bioaccessible**	**B%**	**Total**	**Bioaccessible**	**B%**
Yellow tomato	0.49 ± 0.04 ^a^	0	0.14 ± 0.02 ^a^	0.086 ± 0.001	63.61	0.032 ± 0.004 ^b,c^	0.0087 ± 0.0002	27.48	0.18 ± 0.02 ^b^	0.09 ± 0.01	49.03
Round tomato	0.294 ± 0.001 ^b^	0	0.12 ± 0.01 ^a,b^	0.046 ± 0.005	38.26	0.047 ± 0.005 ^a^	0.0145 ± 0.0007	30.60	0.345 ± 0.001 ^a^	0.060 ± 0.001	17.47
Long tomato	0.279 ± 0.004 ^b^	0	0.122 ± 0.003 ^a^	0.104 ± 0.003	84.82	0.037 ± 0.001 ^b^	0.018 ± 0.001	50.05	0.160 ± 0.006 ^b^	0.059 ± 0.009	37.08
Oxheart tomato	0.19 ± 0.02 ^c^	0	0.085 ± 0.003 ^b^	0.042 ± 0.002	49.51	0.023 ± 0.003 ^c^	0.0187 ± 0.0001	82.40	0.08 ± 0.01 ^c^	0.058 ± 0.006	70.95
One-way ANOVA ^#^	<0.001		0.012		59 ± 18 *	<0.001		48 ± 23 *	<0.001		44 ± 20 *

^#^ In each column, *p* < 0.05 indicates that the mean value of the evaluated parameter of at least one sample differs from the others (significant differences are represented by different letters). B%: percent bioaccessibility; * mean percent bioaccessibility calculated from the results of the four tomato farmers’ varieties; nd: not determined (the utilization of a buffer of NaHCO_3_ during dialysis did not allow us to obtain precise results for Na bioaccessibility).

**Table 3:** Dietary reference intakes (DRIs) for minerals and contribution of the studied tomato farmers’ varieties (average per 100 g portion).

Macromineral	Na			K			Ca			Mg		
	DRI (mg/day)	Contribution to DRI (%)		DRI (mg/day)	Contribution to DRI (%)		DRI (mg/day)	Contribution to DRI (%)		DRI (mg/day)	Contribution to DRI (%)	
		Total Content	Bioaccessible Fraction		Total Content	Bioaccessible Fraction		Total Content	Bioaccessible Fraction		Total Content	Bioaccessible Fraction
Yellow tomato	2000 ^#^	0.15	-	2000	10.76	1.89	800	0.85	0.09	375	2.48	1.36
Round tomato		0.07	-		8.72	1.50		0.55	0.03		2.47	1.19
Long tomato		0.06	-		8.50	1.05		0.56	0.16		2.75	1.37
Oxheart tomato		0.03	-		7.88	1.19		0.63	0.16		2.38	1.49
		0.08 ± 0.05 *	-		9 ± 1 *	1.4 ± 0.3 *		0.6 ± 0.1 *	0.11 ± 0.05 *		2.5 ± 0.1 *	1.4 ± 0.1 *
**Trace** **element**	**Fe**			**Cu**			**Mn**			**Zn**		
	**DRI (mg/day)**	**Contribution to DRI (%)**		**DRI (mg/day)**	**Contribution to DRI (%)**		**DRI (mg/day)**	**Contribution to DRI (%)**		**DRI (mg/day)**	**Contribution to DRI (%)**	
		**Total Content**	**Bioaccessible Fraction**		**Total Content**	**Bioaccessible Fraction**		**Total Content**	**Bioaccessible Fraction**		**Total Content**	**Bioaccessible Fraction**
Yellow tomato	14	3.47	0	1	13.53	8.60	2	1.59	0.44	10	1.78	0.87
Round tomato		2.10	0		12.06	4.62		2.36	0.72		3.45	0.60
Long tomato		1.99	0		12.25	10.39		1.85	0.92		1.60	0.59
Oxheart tomato		1.37	0		8.54	4.23		1.14	0.94		0.82	0.58
		2.2 ± 0.8 *	0 *		12 ± 2 *	7 ± 3 *		1.7 ± 0.5 *	0.8 ± 0.2 *		1.9 ± 0.9 *	0.7 ± 0.1 *

^#^ Level likely to allow most of the general population to maintain Na balance and for which there is sufficient confidence in a reduced risk of CVD in the general adult population [50]. * Mean contribution calculated from the results of the four tomato farmers’ varieties.

**Table 4:** Dietary reference intakes (DRIs) for minerals and contribution of the studied tomato farmers’ varieties (average per tomato fruit unit; see Table 1).

Macromineral	Na			K			Ca			Mg		
	DRI (mg/day)	Contribution to DRI (%)		DRI (mg/day)	Contribution (%)		DRI (mg/day)	Contribution (%)		DRI (mg/day)	Contribution (%)	
		Total Content	Bioaccessible Fraction		Total Content	Bioaccessible Fraction		Total Content	Bioaccessible Fraction		Total Content	Bioaccessible Fraction
Yellow tomato	2000 ^#^	0.28	-	2000	20.45	3.59	800	1.62	0.17	375	4.72	2.64
Round tomato		0.08	-		10.12	1.74		0.64	0.04		2.86	1.38
Long tomato		0.08	-		11.22	1.38		0.74	0.21		3.63	1.81
Oxheart tomato		0.14	-		36.62	5.54		2.92	0.75		11.09	6.95
		0.14 ± 0.08 *	-		20 ± 11 *	3 ± 2 *		1.5 ± 0.9 *	0.3 ± 0.3 *		6 ± 3 *	3 ± 2 *
**Trace** **element**	**Fe**			**Cu**			**Mn**			**Zn**		
	**DRI** **(mg/day)**	**Contribution (%)**		**DRI** **(mg/day)**	**Contribution (%)**		**DRI** **(mg/day)**	**Contribution (%)**		**DRI** **(mg/day)**	**Contribution (%)**	
		**Total content**	**Bioaccessible fraction**		**Total content**	**Bioaccessible fraction**		**Total content**	**Bioaccessible fraction**		**Total content**	**Bioaccessible fraction**
Yellow tomato	14	6.58	0	1	25.70	16.35	2	3.02	0.83	10	3.39	1.66
Round tomato		2.44	0		13.99	5.35		2.74	0.84		4.00	0.70
Long tomato		2.63	0		16.17	13.71		2.44	1.22		2.12	0.79
Oxheart tomato		6.39	0		39.72	19.67		5.28	4.35		3.81	2.70
		5 ± 2 *	0 *		24 ± 10 *	14 ± 5 *		3 ± 1 *	2 ± 1 *		3.3 ± 0.7 *	1.5 ± 0.8 *

^#^ Level likely to allow most of the general population to maintain Na balance and for which there is sufficient confidence in a reduced risk of CVD in the general adult population [50]. * Mean contribution calculated from the results of the four tomato farmers’ varieties.

## Data Availability

Data is contained within the article.
